# Prevention of C5aR1 signaling delays microglial inflammatory polarization, favors clearance pathways and suppresses cognitive loss

**DOI:** 10.1186/s13024-017-0210-z

**Published:** 2017-09-18

**Authors:** Michael X. Hernandez, Shan Jiang, Tracy A. Cole, Shu-Hui Chu, Maria I. Fonseca, Melody J. Fang, Lindsay A. Hohsfield, Maria D. Torres, Kim N. Green, Rick A. Wetsel, Ali Mortazavi, Andrea J. Tenner

**Affiliations:** 1 Department of Pathology and Laboratory Medicine, School of Medicine, University of California, Irvine, CA USA; 2 Department of Developmental and Cell Biology, University of California, Irvine, CA USA; 3 Department of Neurobiology and Behavior, University of California, Irvine, CA USA; 4 Department of Molecular Biology and Biochemistry, University of California, Irvine, CA USA; 5 Research Center for Immunology and Autoimmune Diseases, Institute of Molecular Medicine for the Prevention of Human Diseases, University of Texas-Houston, Houston, TX USA; 6 Present Address: Ionis Pharmaceuticals Inc., Carlsbad, CA 92010 USA

**Keywords:** C5a, C5a receptor, Alzheimer’s disease, Complement, Microglia, Mouse models, Behavior, Gene expression, Inflammation, Phagosome

## Abstract

**Background:**

Pharmacologic inhibition of C5aR1, a receptor for the complement activation proinflammatory fragment, C5a, suppressed pathology and cognitive deficits in Alzheimer's disease (AD) mouse models. To validate that the effect of the antagonist was specifically via C5aR1 inhibition, mice lacking C5aR1 were generated and compared in behavior and pathology. In addition, since C5aR1 is primarily expressed on cells of the myeloid lineage, and only to a lesser extent on endothelial cells and neurons in brain, gene expression in microglia isolated from adult brain at multiple ages was compared across all genotypes.

**Methods:**

C5aR1 knock out mice were crossed to the Arctic AD mouse model, and characterized for pathology and for behavior performance in a hippocampal dependent memory task. CX3CR1^GFP^ and CCR2^RFP^ reporter mice were bred to C5aR1 sufficient and knockout wild type and Arctic mice to enable sorting of microglia (GFP-positive, RFP-negative) isolated from adult brain at 2, 5, 7 and 10 months of age followed by RNA-seq analysis.

**Results:**

A lack of C5aR1 prevented behavior deficits at 10 months, although amyloid plaque load was not altered. Immunohistochemical analysis showed no CCR2^+^ monocytes/macrophages near the plaques in the Arctic brain with or without C5aR1. Microglia were sorted from infiltrating monocytes (GFP and RFP-positive) for transcriptome analysis. RNA-seq analysis identified inflammation related genes as differentially expressed, with increased expression in the Arctic mice relative to wild type and decreased expression in the Arctic/C5aR1KO relative to Arctic. In addition, phagosomal-lysosomal gene expression was increased in the Arctic mice relative to wild type but further increased in the Arctic/C5aR1KO mice. A decrease in neuronal complexity was seen in hippocampus of 10 month old Arctic mice at the time that correlates with the behavior deficit, both of which were rescued in the Arctic/C5aR1KO.

**Conclusions:**

These data are consistent with microglial polarization in the absence of C5aR1 signaling reflecting decreased induction of inflammatory genes and enhancement of degradation/clearance pathways, which is accompanied by preservation of CA1 neuronal complexity and hippocampal dependent cognitive function. These results provide links between microglial responses and loss of cognitive performance and, combined with the previous pharmacological approach to inhibit C5aR1 signaling, support the potential of this receptor as a novel therapeutic target for AD in humans.

**Electronic supplementary material:**

The online version of this article (10.1186/s13024-017-0210-z) contains supplementary material, which is available to authorized users.

## Background

Alzheimer’s disease is a neurodegenerative disorder associated with the loss of cognitive function and characteristic neuropathological changes that include synaptic and neuronal loss, neurofibrillary tangles and extracellular senile plaques composed of amyloid beta (Aß) protein deposits. The association of complement proteins and reactive glia with senile plaques suggests that inflammatory processes may play a role in the disease and that complement activation may contribute to the inflammatory environment [1–5].

Inflammation is a fundamental response to infection and injury. In the brain, the primary immune cells are microglia (reviewed in [6]). In AD, activated microglia have been found to be associated with fibrillar (beta sheet) Aß (fAß) plaques in both human brains and mouse models of AD [7, 8]. Microglia are of particular interest in Alzheimer’s disease since microglial dysfunction has been implicated as a factor in the progression of AD [9]. Indeed, Zhang and colleagues in an unbiased systems approach identified the immune modulatory functions of microglia, such as cytokines, Fc receptors, toll-like receptors (TLRs) and complement to be key nodes and networks of late onset Alzheimer’s disease [10].

The complement system is a well-known powerful effector mechanism of the immune system that normally contributes to protection from infection and resolution of injury [11]. Complement activation generates activation fragments C3a and C5a that interact with cellular receptors to recruit and/or activate phagocytes (including microglia) [12, 13]. While complement activation in the brain can be beneficial and indeed crucial at times, it can also be detrimental. Recently it has been shown that early components of the classical complement pathway are essential for proper synaptic pruning during development [14]. However, excessive complement-dependent synapse elimination seen during aging and in certain neurodegenerative disorders is correlated with loss of, or aberrant, neuronal function [15–18]. Fibrillar Aß can activate the classical complement pathway (in the absence of antibody) or the alternative complement pathway in vitro [19, 20] and in vivo as evidenced by the association of complement components with fAß plaques in human AD and mouse models of AD [2, 21, 22]. Potential consequences of this activation include opsonization (with C3b and/or iC3b) of fAß for engulfment by phagocytes, leukocyte recruitment (by C3a and C5a) and potential bystander lysis on neuronal cells by the membrane attack complex (C5b-9) (reviewed in [23]). In addition, in vitro cultures of C57BL6/J cortical neurons express C5aR1 and, upon binding, C5a is reported to injure neurons [24, 25]. Microglia also express C5aR1 leading to C5a-induced chemotactic functions [26]. In addition, microglia express TLRs. TLR stimulation has been shown to synergize with C5aR1 signaling in the periphery in mice, increasing the pro-inflammatory cytokine production over TLR signaling alone [27, 28]. Thus, strategic therapeutic targeting, such as through pharmacologic treatment with C5aR1 antagonists, could retain the beneficial effects of complement (such as enhanced clearance and neuroprotection) while inhibiting some of the detrimental aspects of complement activation.

Previous studies in our lab have demonstrated that treatment of mouse models of AD with a specific C5aR1 antagonist, PMX205, decreased fibrillar plaque accumulation and microglial CD45 expression and enhanced behavioral performance [7]. To validate C5aR1 as a therapeutic target, we investigated the effect of C5aR1 gene ablation on behavior, pathology and microglial gene expression in wild type and the Arctic AD mouse model. The Arctic mouse model has the human APP transgene containing three mutations (Swedish, Indiana, and Arctic) that increase risk of AD in humans [29]. The Arctic mutation results in the increased rate of fibril formation upon generation of the cleaved Aß42 peptide, resulting in a quite aggressive rate of fibrillar amyloid plaque deposition, initiating as early as 2 months of age in these mice [30, 31]. Since the fibrillar form of Aß is known to activate complement, C5a should be generated. Therefore, we crossed Arctic mice [30] with C5aR1KO [32] mice to test the hypothesis that the absence of C5aR1 would suppress microglial inflammatory responses and behavior deficits. Subsequently, both Arctic and Arctic/C5aR1KO mice were crossed to the CX3CR1^GFP^ and CCR2^RFP^ reporter mice [33, 34] to enable fluorescence-activated cell sorting (FACS) of adult microglia (GFP+ RFP-) from monocytes/macrophages (GFP+ RFP+) isolated from brain at different ages for microglial specific gene expression analysis.

Our findings demonstrate that deletion of C5aR1 prevents the loss of neuronal complexity in hippocampal CA1 and protects from spatial cognitive deficits even in the presence of fibrillar amyloid plaques. Moreover, microglial transcriptome analysis revealed increased expression of inflammatory genes, including chemokines and members of the NF-κB pathway, in the Arctic mouse as early as 5 months of age that remained elevated through 10 months of age but were not so upregulated in the Arctic/C5aR1KO. Additionally, clearance and degradation pathways, such as the phagosome and lysosome pathways, were increased in the Arctic/C5aR1KO relative to the Arctic mice as early as 2 months of age and persisted through 10 months. Taken together, the data suggest that a lack of C5aR1 signaling prevents the C5a-induced polarization to more inflammatory, less phagocytic microglia. The absence of C5a-C5aR1 signaling also protected from cognitive deficits. The data supports further investigation of the use of antagonists for C5aR1 as part of a novel therapeutic strategy to slow the progression of Alzheimer’s disease cognitive decline.

## Methods

### Animals

All animal experimental procedures were reviewed and approved by the Institutional Animal Care and Use Committee of University of California at Irvine, and performed in accordance with the NIH Guide for the Care and Use of Laboratory Animals. The AD mouse model used was the Arctic48, which carry the human APP transgene with the Indiana (V717F), Swedish (K670 N + M671 L), and Arctic (E22G) mutations (under the control of the platelet-derived growth factor-ß promoter), and thus produce Aß protofibrils and fibrils as early as 2–4 months old [30], graciously provided by Dr. Lennart Mucke (Gladstone Institute, San Francisco, CA, USA). C5aR1 knockout mice generated by target deletion of the C5a receptor gene [32], were crossed with Arctic^+/−^ mice to produce Arctic mice lacking C5aR1 (Arctic/C5aR1KO) and wild type littermate mice lacking the C5a receptor (C5aR1KO) that were assessed for behavioral deficits and pathology in comparison to the C5aR1 sufficient animals. To enable isolation of microglia distinct from any infiltrating myeloid cells for differential gene expression studies, homozygous breeding pairs of CX3CR1-GFP and CCR2-RFP mice were obtained from Jackson Laboratories and bred to produce double homozygous reporter mice (CX3CR1^GFP/GFP^CCR2^RFP/RFP^). Double homozygous reporter mice were bred with Arctic model mice to generate Arctic^+/−^CX3CR1^GFP/+^CCR2^RFP/+^ (Arctic) and littermates lacking the APP transgene that were used as controls (WT). CX3CR1^GFP/GFP^CCR2^RFP/RFP^ mice were bred to C5aR1 knockout to produce CX3CR1^GFP/GFP^CCR2^RFP/RFP^ mice lacking C5aR1. Then these mice were bred to Arctic+/− mice lacking the C5a receptor (Arctic/C5aR1KO), to generate wild type and Arctic/C5aR1KO all heterozygotic for CX3CR1^GFP^ and CCR2^RFP^. None of the mice generated exhibited any gross abnormalities. Both males and females were used in all experimental assessments.

### Behavior testing

Mice were handled for two minutes each for five consecutive days prior to all behavior testing. After handling, mice were acclimated (allowed to explore) to a behavior chamber for five minutes/day, for four consecutive days. On the training day (24 h following the last day of habituation), mice were exposed to two of the same objects (e.g. 100 mL glass beakers, blue legos, or opaque light bulbs) in fixed positions within the context chamber near one wall. Twenty-four hours later mice were tested in a hippocampal independent novel object recognition task (iNOR), or in a hippocampal dependent object location memory task (OLM). The iNOR paradigm involved exchanging one of the objects for a novel object (e.g. blue lego or 100 mL beaker). Testing for spatial memory (OLM) was performed by moving one of the objects to the center of the context, thus changing the spatial relationship of that object within the context. Animals’ preference for novelty was tested by determining the time spent with familiar and novel objects or locations. Objects used, sizes of objects, and dimensions of the contexts were as described [35]. To prevent olfactory distractions, all objects were cleaned with ethanol and the bedding stirred after each trial. Testing was scored blind by two independent observers using stopwatches and results correlated. There were no significant differences found in performance of mice of any of the genotypes during habituation to the context, using ANY-maze video tracking system software (version 4.82, Stoelting Co.) to assess video recordings on Day 4 (the day preceding training) during which speed, distance traveled, % time spent in an inner zone of the chamber, and % time spent in an outer zone of the chamber were scored (see Additional file 1). This demonstrates that there were no motor or motivational impairments in the different genotypes. Data from behavior performance were converted with the formula (time spent with novel object minus time spent with familiar object)/time spent with both objects) × 100 to obtain a percent (%) discrimination index [36]. Data are presented as mean +/− SEM. Results were compared with two-tailed non-parametric Mann-Whitney *U* test or unpaired t-test or ANOVA followed by Tukey’s post hoc tests. Differences were considered significant when p was < 0.05. Mice were removed from the analysis if they spent less than 1 s/min with the objects during training and/or testing, or if the performance of mice was +/− 2 standard deviations from the mean.

### Microglia isolation and FACS

Adapted from [37], mice from WT, C5aR1KO, Arctic and Arctic/C5aR1KO genotypes were perfused with PBS at 2, 5, 7 and 10 months, and the brains (without olfactory bulbs, cerebellum, or midbrain) collected for microglia isolation. Brains were treated with Dispase II (Roche) at 37 °C for 1 h and passed through a 70 μM nylon cell mesh strainer (BD Biosciences) to obtain a single cell suspension. The cell suspension was washed in Hanks’ balanced salt solution (HBSS; ThermoFisher) and spun at 1000 x g for 10 min at 4 °C. The cell pellet was resuspended in 70% Percoll (Sigma; diluted in HBSS), and 35% Percoll (diluted in HBSS) was carefully layered over to create a discontinuous Percoll gradient. After centrifugation at 800 x g for 45 min at 4 °C, the cells at the interphase were quickly collected, washed with HBSS +2% heat-inactivated fetal bovine serum (Gibco) and passed through a 40 μM nylon mesh cell strainer. Using a FACS Aria II, the viable cell population was gated based on forward and side scatter properties of a propidium iodide stained control. Using these forward and side scatter parameters, the GFP^+^RFP^+^ and the GFP^+^RFP^−^ cells were sorted and collected directly into RA-1 lysis buffer (Illustra RNAspin mini isolation kit, GE Healthcare).

### Immunohistochemistry

Some cohorts of mice were perfused with phosphate buffered saline (PBS). The brain was dissected at 4 °C with half of the tissue immediately frozen on dry ice for biochemical analysis, and the other half drop-fixed in 4% paraformaldehyde-PBS at 4 °C for immunohistochemistry. After 24 h, the fixed tissue was placed in PBS/0.02% sodium azide at 4 °C until use. Vibratome sections (coronal, 40 μm) were incubated sequentially with 3%H_2_O_2_/10% Methanol/Tris buffer saline (TBS) to block endoperoxidase and with 2% BSA/0.1%Triton X-100/TBS to block nonspecific binding. Tissues were incubated overnight at 4 °C with anti mouse CD45 antibodies (rat monoclonal anti CD45, 1 μg/mL, Serotec, or goat anti CD45 0.2 μg/mL, R&D systems, Minneapolis, MN), anti Aß antibody (rabbit polyclonal 1536 [38] gift from N.R Cooper) or appropriate control IgG in blocking solution. Primary antibodies were detected with biotinylated secondary antibodies against the corresponding species, followed by ABC complex and DAB (VECTOR, Burlingame, CA). In animals lacking GFP constructs, fibrillar Aß was stained with 1% thioflavine as previously described [39]. For immunofluorescence staining of total Aß, samples were incubated with blocking solution followed by rabbit polyclonal anti Aß (1536) and labeling detected with Alexa 405 anti rabbit secondary antibody (1:300, Invitrogen). For Iba1/Thioflavine double label, sections were washed with 50% ethanol for 3 min., incubated with 0.5% thioflavine for 10 min, and after washing were blocked with PBS + 0.2% TritonX-100 + 5% goat serum for 1 h and subsequently incubated with anti IBA1 (Wako, I μg/ml in blocking buffer) 4 °C overnight. After washing, anti-rabbit Alexa 647 (1:200, Invitrogen) was added for one hour room temperature.

Stained sections were observed under a Zeiss Axiovert-200 inverted microscope (Carl Zeiss, Thornwood, NY) and images were acquired with a Zeiss Axiocam high-resolution digital color camera (1300 × 1030 pixels) and analyzed using Axiovision 4.6 software. Percent of immunopositive area (% Field Area) (immunopositive area / total image area × 100) was determined for all the markers studied by averaging 2–4 images per section for Aß and thioflavine or 6 images per section for CD45 of the hippocampal area. Digital images were obtained using the same settings, and the segmentation parameters were constant as previously reported [40]. The mean value of the % Field Area for each marker in each animal was averaged per genotype group with the number of animals per group indicated in figure legends. Double labelled IBA1 and thioflavine images were collected on Leica DM5500 Q Confocal Microscope. IBA1+ microglial cell numbers and ThioS+ plaque numbers and sizes were determined using the spots and surfaces modules in Bit-plane Imaris 7.5 software, respectively. Data were analyzed using one-way ANOVA statistical analysis. Staining of all genotypes that were compared by image analysis in an experiment was done simultaneously per given marker.

### Golgi staining and Sholl analysis

Mice were perfused as above, and half brains were fixed and, after sectioning (150 μm), stained using the superGolgi Kit (Bioenno Lifesciences, Santa Ana, CA), following the instructions of the manufacturer. All sections were coded, such that the analysis was done blinded. The stained tissues were then quantified using a stereology Zeiss Axio Imager M2 with NeuroLucida software Version 11.03. The soma and apical dendrites along with its dendritic branching were traced under 100× magnification for 5 pyramidal neurons in the CA1 region of the hippocampus for each animal followed by Sholl analysis, determining the average number of branching for a given distance from the soma (μm) of each neuron. The average number of branchings for a given distance from the soma for the neurons of each animal and genotype +/− SEM were generated using GraphPad Prism. Significance is defined as *p* < 0.05.

### RNA extraction and RNA-Seq

Total RNA from GFP^+^RFP^−^ cells was extracted using the Illustra RNAspin mini isolation kit. RNA was quantified using the NanoDrop ND-1000 spectrophotometer (ThermoFisher) and the quality checked using the Agilent Bioanalyzer 2100 (Agilent Technologies). Sequencing was performed by the University of California at Irvine Genomics High Throughput Facility. The SMARTer Stranded Total RNA-Seq Kit - Pico Input Mammalian (Clontech Laboratories) was used to generate Illumina-compatible RNA-seq libraries. Briefly, total RNA was converted into cDNA and then adapters with barcodes for Illumina sequencing were added by PCR. The PCR products were then purified and the ribosomal cDNA depleted with sequence specific probes and RNaseH. The resulting cDNA fragments were further amplified (12 cycles) and the PCR products were purified once again to yield the final cDNA libraries. The barcoded cDNA libraries were multiplexed on the Illumina HiSeq 2500 platform to yield 100-bp paired-end reads.

### Read alignment and expression quantification

Pair-end RNA-seq reads were aligned using STAR v.2.5.1b [41] with parameters ‘--outFilterMismatchNmax 10 --outFilterMismatchNoverReadLmax 0.1 --outFilterMultimapNmax 10’ to the reference genome GRCm38/mm10. We used STAR to then convert to transcriptome-based mapping with gene annotation Gencode v.M8. Gene expression was measured using RSEM v.1.2.25 [42] with expression values normalized into transcripts per million (TPM).

### Building of gene expression matrix for time-course gene expression and pathway analysis

Libraries with uniquely mapping percentages higher than 65% were considered to be of good quality and kept for downstream analysis. Protein coding and long non-coding RNA genes, with expression TPM > =0.5 in at least one of the duplicates for each genotype, were collected and quantile normalized for subsequent time-course and pathway analysis. Fastq files and processed data matrices were deposited in GEO with the accession ID GSE93824.

### Time-course gene expression analysis

Quantile normalized gene expression (TPM) profiles were analyzed with maSigPro [43] to identify distinct gene expression profiles across time points whose expression differed in at least one other age in one other genotype with multiple cluster sizes. A cluster size of k = 9 gave the best set of profiles. An alpha of 0.05 for multiple hypothesis testing and r^2^ = 0.7 for regression model were used to acquire significant genes for each cluster.

### Gene ontology and pathway analysis

Gene clusters were analyzed for Gene ontology (GO) enrichment by Metascape (http://metascape.org) [44] using a hypergeometric test corrected *p*-value lower than 0.05. maSigPro clusters were also analyzed for KEGG pathway enrichments using PaintOmics 3 [45] with significant pathways selected based on Fisher’s exact test p-value lower than 0.05.

### qRT-PCR

RNA was converted to cDNA using Superscript III reverse transcriptase (Life Technologies) following manufacturer’s instructions and quantified using the NanoDrop ND-1000 spectrophotometer (ThermoFisher). Quantitative RT-PCR was performed using the StepOnePlus Real-Time PCR System, the StepOne software v2.3 (Applied Biosystems) with the following FAM dye and MGB quencher TaqMan probes (ThermoFisher): Mm00443111_m1 (Ccl4), Mm00436450_m1 (Cxcl2), Mm00437306_m1 (Vegfa), Mm00515586_m1 (Ctsd), Mm00487585_m1 (Man2b1, Mm00499230_m1 (Npc2). All genes were multiplexed with the VIC dye and MGB quencher probe Mm01545399_m1 (Hprt) to allow for normalization of housekeeping gene in the same well. For each gene, cDNA from three samples (unique animals) of each genotype was tested in triplicate. Two of the samples were from the same samples as the RNA-seq dataset and one was from a sample not analyzed by RNA-seq at ages 5 and 10 months. *P* values are calculated using one way ANOVA followed by Sidak’s multiple comparison test.

## Results

### C5aR1 deficiency prevents deficits in spatial specific object location memory

**Fig. 1:**
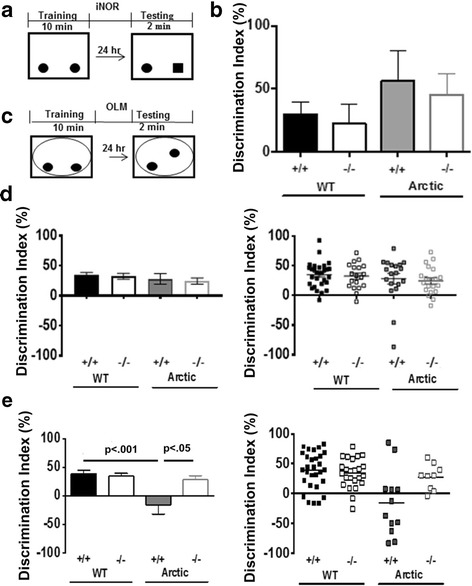
Performance in Novel Object and Object Location Memory Tasks. Animals were tested in novel object recognition test (**a,b**) and novel object location memory test (**c-e**) after 5 days of handling and 4 days of habituation to the testing chamber. Paradigm schematics are presented for iNOR (**a**) and OLM (**c**). Solid oblongs or circles in the boxes represent identitcal objects. After Day 1 training, one familiar object was replaced with a novel object (solid square) (**a**) or a familiar object was moved to the center of the context (**c**). The novel object replacement or the object moved was counter balanced among all trials. Performance was evaluated using the first two minutes of testing at 9 months for iNOR (**b**), and either 7 months (**d**) or 10 months (**e**) for OLM. (**b**) WT *n* = 11, C5aR1KO *n* = 6, Arctic *n* = 4, Arctic/C5aR1KO *n* = 5. Right plots in **d** and **e** are the scatter plots for left bar graphs, respectively. (**d**) *n* = 29 (WT), 19 (C5aR1KO), 19 (Arctic) and 20 (Arctic/C5aR1KO). (**e**) *n* = 29 (WT), 25 (C5aR1KO), 12 (Arctic) and 9 (Arctic/C5aR1KO). (**b,d,e**) Data is expressed as the average +/− SEM. *P* values were determined by ANOVA followed by Tukey’s post hoc test

Since a C5aR1 antagonist had previously been shown to suppress AD-like pathology in mouse models of AD [7], we evaluated the effect of C5aR1 gene ablation on the ability to perform memory tasks. Spatial memory cognitive deficits are observed in AD patients and the Arctic AD mouse model has been shown to recapitulate specifically hippocampal dependent spatial impairments [31]. Our hypothesis was that knocking out C5aR1 would lead to less inflammation and, thus, to better performance. To confirm that the behavior deficit is spatial and hippocampal-dependent we used both the traditional hippocampus independent novel object recognition (iNOR) and a modification of the iNOR paradigm that assessed spatial memory and was hippocampal dependent, the object location memory (OLM) task [46, 47]. Mice of all 4 genotypes (WT, C5aR1KO, Arctic, Arctic/C5aR1KO) spent about 50% of their time with each object during the training phase demonstrating no preference for a certain side of the chamber or one of the objects within the chamber indicating that memory retrieval 24 h later would not be confounded by inherent spatial or object preferences (Additional file 1E). As expected, at 7 (data not shown) and 9 (Fig. 1a,b) months of age, all genotypes performed well, and there were no differences in performance between genotypes in the hippocampal independent iNOR task demonstrating that the Arctic mice as well as C5aR1 deficient mice were not impaired.

When tested in the spatial OLM task (Fig. 1c), WT and C5aR1KO mice at both 7 and 10 months of age exhibited a discrimination index greater than 30% during the first two minutes of testing, indicating that they had a strong preference for exploring the object in the new location and that genetic deletion of C5aR1 alone is not detrimental or beneficial (Fig. 1d,e). The C5aR1 sufficient Arctic mice did not exhibit significant behavioral deficits at 7 months, although there were 2 animals that were noticeably performing worse than all other mice (Fig. 1d, right). However, at 10 months of age, there was a significant deficit (*p* < .001) in performance of Arctic mice in comparison to wild type mice, while importantly, Arctic/C5aR1KO did not differ from wild type or C5aR1KO. A statistically significant difference in behavior (*p* < .05) was observed between the Arctic mice lacking C5aR1 compared to the C5aR1 sufficient Arctic mice indicating protection from deficit in the Arctic/C5aR1KO mice relative to the Arctic mouse (Fig. 1e), consistent with the hypothesis that blocking C5aR1-mediated events prevents or slows the progression of cognitive defects.

### Plaque accumulation and microglia marker CD45 immunoreactivity are not necessarily indicative of cognitive loss

**Fig. 2:**
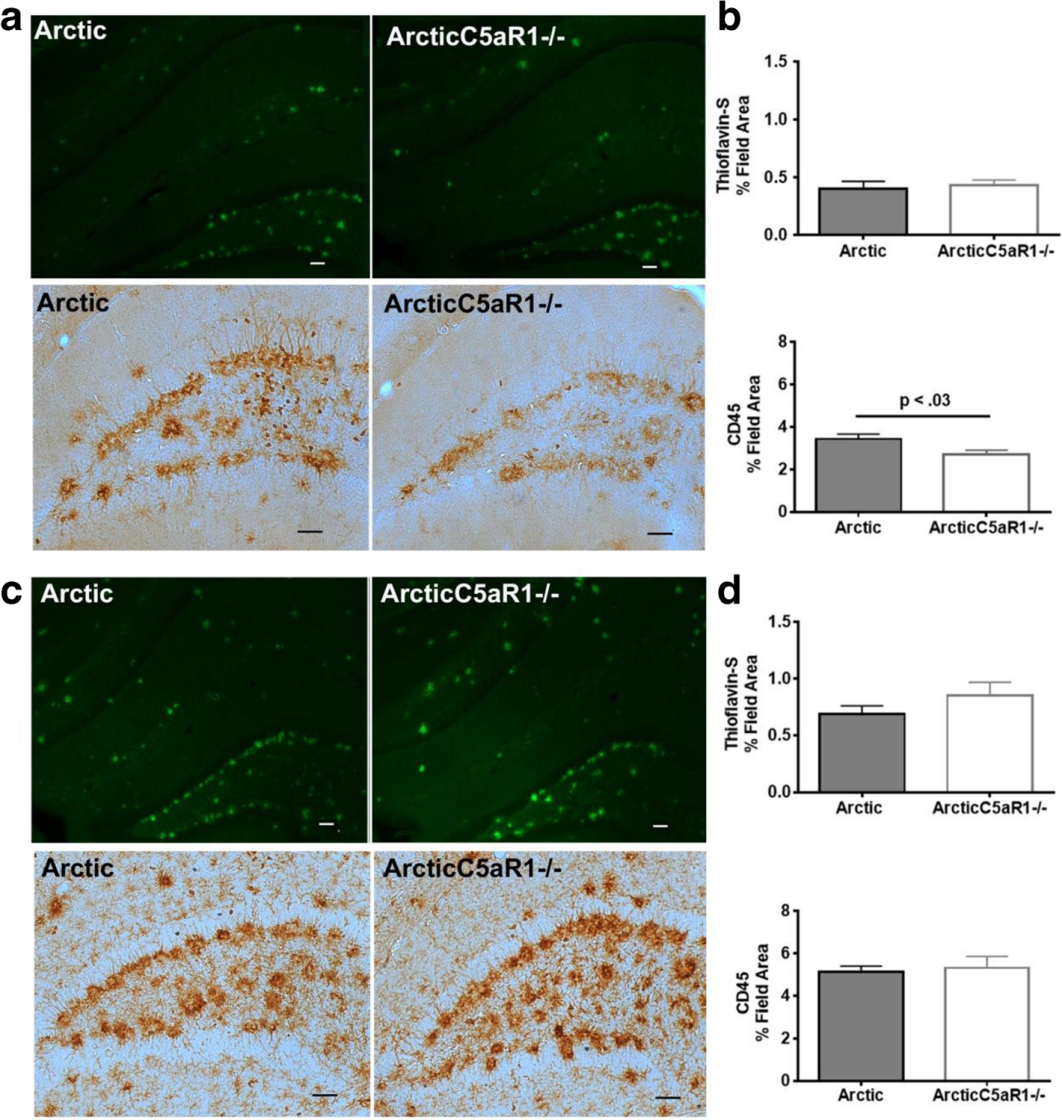
Fibrillar plaque and CD45 microglial expression in hippocampus. Representative pictures of thioflavine-S staining (fibrillar plaques) (top panels) and CD45 staining (microglia) (lower panels) at 7 (**a**) and 10 months (**c**). Scale bar: 50 um. (**b**) Image analysis of 7 month thioflavine-S (upper) and CD45 (lower) staining (Arctic *n* = 9, Arctic/C5aR1KO *n* = 9) (**d**) Image analysis of 10 mo thioflavine (Arctic *n* = 12, Arctic/C5aR1KO *n* = 9) (upper) and CD45 (Arctic *n* = 11, Arctic/C5aR1KO *n* = 7) lower). Bars represent group means +/− SEM of *n* animals per genotype. Data represent the average of 2 to 4 sections per animal. P value was calculated using one- way ANOVA

Arctic mice at 7 and 10 months of age have abundant fibrillar plaque pathology in the hippocampus. Genetic deletion of C5aR1 did not alter the extent of fibrillar plaque pathology at either 7 or 10 months of age (Fig. 2; Additional file 2). IBA1 is a myeloid/ microglia specific marker in the brain. While CD45 is expressed on other cell types (T cells and endothelial cells), in the brain microglia are the dominant cells expressing this molecule, and it is also upregulated upon activation. Quantitative image analysis of these two markers revealed a slight but statistically significant decrease in CD45 immunoreactivity at 7 m in the Arctic/C5aR1KO mice compared to the C5aR1 sufficient Arctic mice (20%, *p* = .03) (Fig. 2b), while the number of IBA1 microglia was slightly greater (Additional file 2). However, those differences were not sustained in the 10 month mice (Fig. 2d; Additional file 2). Furthermore, neither fibrillar plaque pathology nor CD45 correlated with behavioral parameters of individual animals (data not shown). These data suggest that neither plaque load nor induction of CD45 is sufficient for neurotoxic effects that impact behavior in this model.

### Lack of CCR2-positive infiltrating monocytes around plaques

In the Arctic AD mouse model, we used the CX3CR1^GFP^ and CCR2^RFP^ reporter mice to differentiate microglia from infiltrating CCR2-positive monocytes [48]. Crossing CX3CR1^GFP/GFP^CCR2^RFP/RFP^ mice with Arctic or Arctic/C5aR1KO mice ultimately generated CX3CR1^+/GFP^CCR2^+/RFP^Arctic^−/−^ (WT) and CX3CR1^+/GFP^CCR2^+/RFP^Arctic^+/−^ (Arctic), CX3CR1^+/GFP^CCR2^+/RFP^C5aR1^−/−^(C5aR1KO) and CX3CR1^+/GFP^CCR2^+/RFP^Arctic^+/−^C5aR1^−/−^ (Arctic/C5aR1KO) mice. While some studies with Alzheimer’s disease mouse models have suggested that CX3CR1 and CCR2 play a role in the progression of disease [49–51], we determined that having a single copy of either gene does not alter plaque deposition or microglial CD45 reactivity (Additional file 3 A-E).

**Fig. 3:**
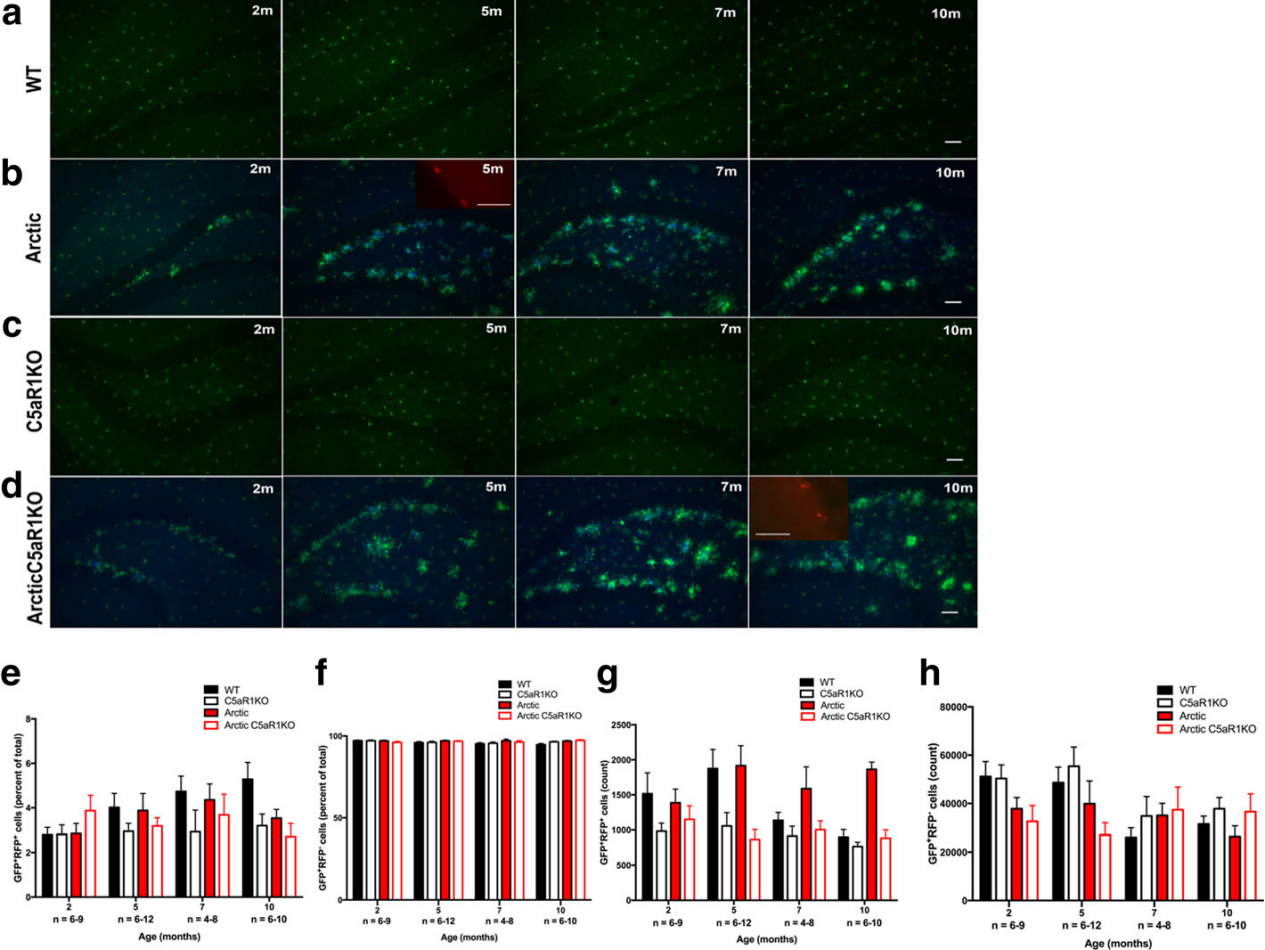
Glia clustering around plaques are CX3CR1^+^CCR2^−^. Brain sections of (**a**) WT, (**b**) Arctic, (**c**) C5aR1KO, and (**d**) Arctic/C5aR1KO mice (all CX3CR1^+/GFP^CCR2^+/RFP^) at 2, 5, 7, and 10 months were fluorescently labelled in blue with anti-Aß antibody (1536). Staining was performed at separate times for cohort one (**a,b**) and cohort 2 (**c,d**). Representative pictures of cohorts one and two were taken at a close but slightly different bregma levels. Inset shows CCR2^+/RFP^ macrophages. Scale bar:50 um (**e**) Percoll purified brain cells from WT, Arctic, C5aR1KO and Arctic/C5aR1KO mice (all CX3CR1^+/GFP^CCR2^+/RFP^; n = 4–12 per genotype, per age) were FACS-sorted as GFP^+^RFP^+^ (**e,g**) and GFP^+^RFP^−^ cells (**f,h**) and quantified as a percentage of total GFP+ cells (**e,f**) and total cell count (**g,h**)

We next proceeded to age wild type (WT), C5aR1KO, Arctic, and Arctic/C5aR1KO mice to 2, 5, 7, and 10–11 months. As expected, no plaque pathology nor microglia clustering was observed in wild type mice or C5aR1KO mice (Fig. 3a,c). Plaque deposition increased with age and microglia clustered around the plaques, but was similar in the Arctic and Arctic/C5aR1KO mice across the different ages (Fig. 3b,d). Strikingly, no RFP-positive cells were found in areas near the plaques at any age. No RFP-positive cells were found anywhere in the parenchyma, while a few RFP-positive cells were localized around the surface of the brain, possibly meningeal macrophages (Fig. 3b,d insets).

Percoll gradient isolated cells, from brains of mice in the same cohort as that analyzed by IHC, when sorted by RFP and GFP showed a small population of GFP and RFP-positive cells. RFP-positive cells (which could represent the meningeal macrophages) represented 2–6% of the total sorted GFP-positive population (Fig. 3e), while RFP-negative microglia made up 94–98% of the GFP-positive cells (Fig. 3f). The absolute number of cells recovered varied, with means among all genotypes and ages between 700 and 2000 for RFP-positive cells (Fig. 3g) and between 27,000 and 55,000 for RFP-negative GFP-positive microglia (Fig. 3h).

### Analysis of age dependent patterns of gene expression

**Fig. 4:**
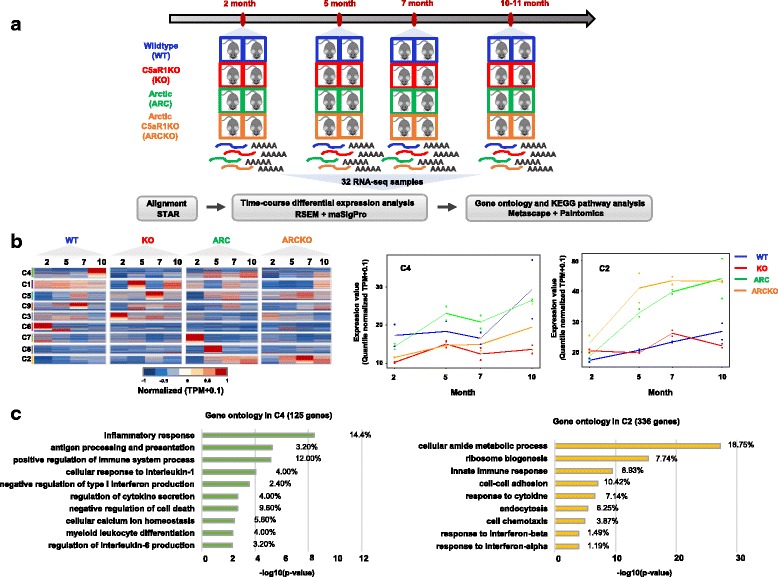
Temporal gene expression profiles across four genotypes. (**a**) RNA-seq experiments were performed using duplicate samples from WT (blue), C5aR1KO (red), Arctic (green) and Arctic/C5aR1KO (orange) mice at 2, 5, 7 and 10–11 months. A total of 32 RNA-seq libraries were sequenced and analyzed. Reads were aligned to the reference genome with STAR. Gene expression level was estimated by RSEM and analyzed with maSigPro to identify distinct gene expression profiles across time. Differentially expressed genes during the time-course were loaded into Metascape and PaintOmics 3 to acquire gene ontology (GO) and KEGG pathways enrichment. (**b,** left) Heatmap of 2156 differentially expressed genes across four genotypes and four ages. Nine gene expression clusters were derived from maSigPro and labeled with number and color on the side. Quantile normalized TPM plus 0.1 were transformed between −1 and 1 to present high (red) and low (blue) expression level within each cluster. (**b**, right) Gene expression profiles for cluster 4 and 2. Median profile are plotted across all replicates for all the genes in the corresponding cluster. WT (blue), C5aR1KO (red), Arctic (green) and Arctic/C5aR1KO (orange). (**c**) Gene ontology enrichment was measured on genes from cluster 4 (green) and 2 (yellow). Gene ontology terms were sorted based on *p*-value. The percentage of genes in the cluster that are contained in the GO term is listed to the right of each bar

To identify transcriptome changes in microglia in Arctic/C5aR1KO mice relative to Arctic mice, the FACS-sorted GFP-positive/RFP-negative cells of WT, C5aR1KO, Arctic, and Arctic/C5aR1KO mice at 2, 5, 7, and 10 or 11 months of age were collected and RNA extracted for RNA-seq analysis (Fig. 4a). RNA quality and quantity was sufficient for RNA sequencing and analysis (Additional file 4). RNA-seq libraries that passed our quality filters were used to estimate gene expression level. By doing time-course analysis with maSigPro [43] we identified 2156 differentially expressed genes that showed dynamic expression patterns over time with age and disease progression. The genes were partitioned into nine clusters, ranging in size from 125 genes to 445 genes, to show distinct temporal and genotype-specific profiles (Fig. 4b, Additional file 5). To identify clusters of interest to study further, we did the following: 1) Assessed the gene expression patterns of each cluster (generated by maSigPro) to identify differences in gene expression between the Arctic and Arctic/C5aR1KO (Fig. 4b, Additional file 5). 2) Performed gene ontology (GO) enrichment analysis using Metascape [44] to aid in the identification of clusters that had genes associated with biologically relevant processes (Additional file 6). 3) Used PaintOmics 3 for pathway analysis to identify significantly enriched KEGG pathways in each cluster (Additional file 7). Cluster 4 contained 125 genes (Additional file 8) that increased with age starting by 5 months in the Arctic relative to wild type. Those genes did not increase in the Arctic/C5aR1KO until 10 months of age and at that time showed levels similar to that seen in the 10 month wild type animals and still lower than the Arctic expression at 10 months (Fig. 4b). Cluster 2 consisted of 336 genes (Additional file 8) that were generally unchanged in the C5aR1KO relative to wild type but increased in the Arctic after 2 months relative to WT. The same genes were further increased in the Arctic/C5aR1KO (Fig. 4b). Gene ontology enrichment analysis using Metascape identified the enriched GO terms for cluster 4 of which the top 3 were: inflammatory response, antigen processing and presentation, and positive regulation of immune system process (Fig. 4c, left), whereas the top 3 enriched GO terms in cluster 2 were cellular amide metabolic process, ribosome biogenesis, and innate immune response (Fig. 4c, right).

### Pathway analysis reveals less inflammation and higher degradative capacity in Arctic/C5aR1KO

**Fig. 5:**
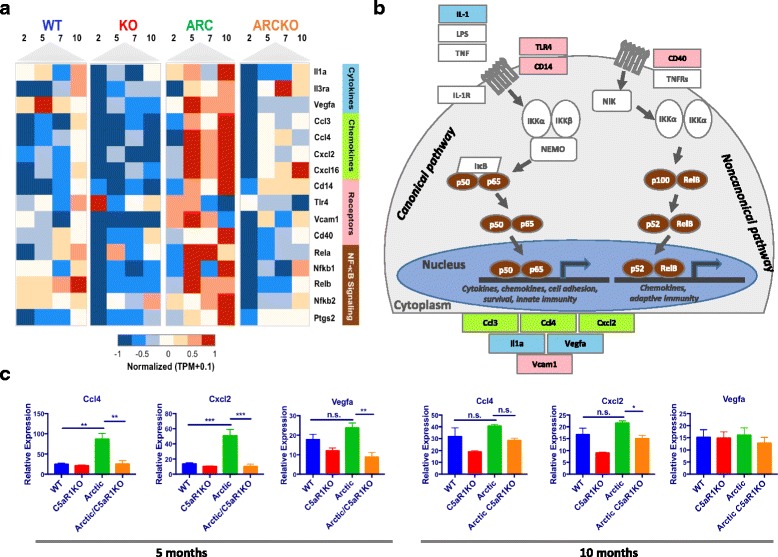
Gene expression profiles of selected genes in inflammation pathways. All genes in heat map were present in cluster 4 and found in the NF-kB signaling pathway, cytokine-cytokine receptor interaction, and/or chemokine signaling KEGG pathways. (**a**) Selected genes were clustered based on molecule functions: cytokines (sky blue), chemokines (yellow green), receptors (pink), NF-kB signaling (brown). Quantile normalized TPM plus 0.1 were transformed between −1 and 1 to present high (red) and low (blue) expression level during time-course across four genotypes for each gene. (**b**) Schematic of the NF-kB pathway with genes present in heat map colored accordingly. (**c**) RT-PCR analysis on a subset of genes to validate the RNA-seq dataset. For each gene, 3 samples, two from the RNA-seq dataset and an additional sample not analyzed by RNA-seq, were analyzed for each genotype at ages 5 (left) and 10 months (right). Bars represent group mean expression +/− SEM. P value was calculated using one way ANOVA followed by Sidak’s multiple comparison test

Pathway analysis of cluster 4 identified several inflammatory diseases pointing to inflammation as well as canonical inflammation pathways. Of interest were NF-κB signaling, cytokine-cytokine receptor interaction, TLR signaling and chemokine signaling due to their statistical significance as well as their biological significance in the context of AD (Additional file 7). These four pathways were then investigated further and the 16 differentially expressed genes between the Arctic and Arctic/C5aR1KO (found in these 4 pathways) were plotted on a heat map (Fig. 5a). These genes remained elevated in the Arctic relative to WT, C5aR1KO, and Arctic/C5aR1KO through 10 months of age (Fig. 5a). Increases in those genes appeared in both WT and Arctic/C5aR1KO only beginning at 10 months (consistent with the increase in inflammatory gene expression with age [52]) but remained significantly lower than in microglia from the Arctic mice. Most of the inflammatory genes shown in Fig. 5a were either NF-κB subunits, transcriptionally regulated by NF-κB or were upstream activators of NF-κB as shown color-coded in Fig. 5b. These differentially expressed genes included cytokines and growth factors or their receptors (*Il1a, Vegfa, Il3ra*), chemokines (*Ccl3, Ccl4, Cxcl2, Cxcl16*), signaling receptors (*Tlr4, Vcam1, Cd40*), NF- κb transcription factor subunits (*Rela, Nfkb1, Relb, Nfkb2) and Ptgs2* (COX2) (Fig. 5a). Representative genes were validated by RT-PCR for each genotype at ages 5 and 10 months. Ccl4 and Cxcl2 were both significantly increased in the Arctic relative to WT at 5 months (3.5× and 4.4× respectively) and were decreased in the Arctic/C5aR1KO by 35% and 23%, respectively relative to the Arctic. Although Vegfa was not significantly increased in the Arctic relative to the WT at 5 months, it was significantly decreased by 46% in the Arctic/C5aR1KO relative to the Arctic (Fig. 5c, left). At 10 months, only Cxcl2 was significantly decreased in the Arctic/C5aR1KO relative to the Arctic and all 3 genes validated by RT-PCR showed no difference in expression between the WT and Arctic (Fig. 5c, right). These data thus follow the same trend observed in the RNA-seq data where C5aR1 deficiency in the Arctic leads to reduced expression of inflammatory genes early in the progression of the disease. Aside from cluster 4, cluster 3 also included some of the same pathways as in Fig. 5, such as chemokine signaling and cytokine-cytokine receptor interaction (Additional file 7), however the genes in this cluster did not contain differences between the Arctic and Arctic/C5aR1KO, rather, the C5aR1KO mice were substantially upregulated relative to the other genotypes.

**Fig. 6:**
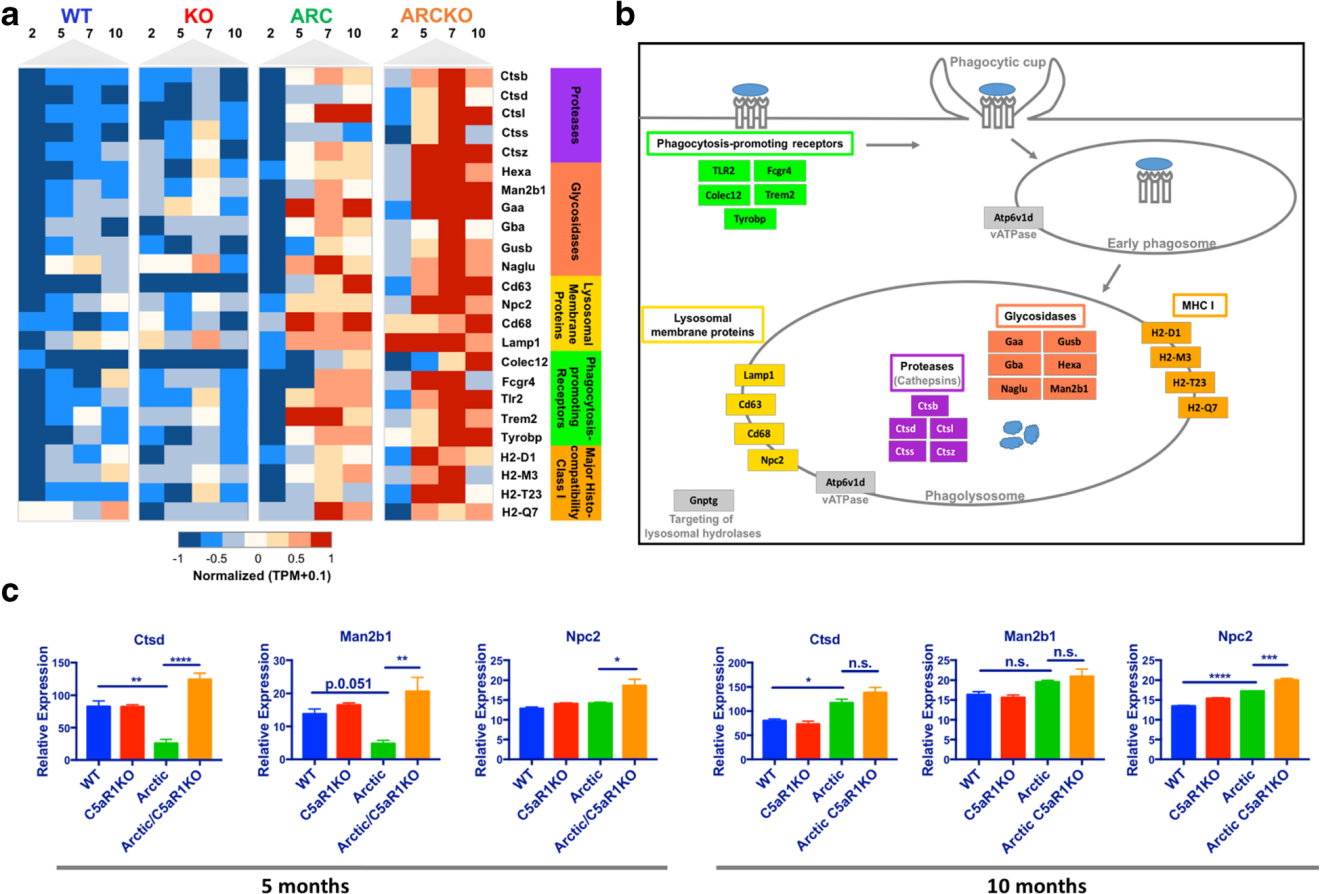
Gene expression profiles of selected genes in lysosome and phagosome pathways. All genes in heat map were present in cluster 2. All but Trem2 and Tyrobp were also found in the phagosome or lysosome KEGG pathways. Trem2 and Tyrobp were added due to known roles in microglial phagocytosis. (**a**) Selected genes were clustered based on molecule functions: protease (purple), glycosidases (coral), lysosomal membrane proteins (yellow), phagocytosis-promoting receptors (green) and major histocompatibility class I (orange). Quantile normalized TPM plus 0.1 were transformed between −1 and 1 to present high (red) and low (blue) expression level during time-course across four genotypes for each gene. (**b**) Schematic of the phagolysosome pathway with genes present in heat map colored accordingly. (**c**). RT-PCR analysis on a subset of genes to validate the RNA-seq dataset, as described in Fig. 5 legend. Bars represent group mean expression +/− SEM (*n* = 3). P value was calculated using one way ANOVA followed by Sidak’s multiple comparison test

Like cluster 4, pathway analysis of cluster 2 genes identified pathways that were of statistical significance as well as biologically relevant for microglia. The lysosome, antigen processing and presentation, and phagosome pathways identified 22 genes that were differentially expressed between the Arctic and Arctic/C5aR1KO. Trem2 and Tyrobp, also included in cluster 2, were added to the heat map due to known roles in microglial phagocytosis (Fig. 6a). In general, the genes in these pathways increased in the Arctic relative to WT at 5 mo but were further increased in the Arctic/C5aR1KO relative to Arctic (and as early as 2 months of age) and generally remained elevated through 10 months (Fig. 6a). The genes included phagocytosis-promoting receptors (*Tlr2, Fcgr4, Colec12, Trem2, Tyrobp*), lysosomal membrane proteins (*Lamp1, Npc2*), cathepsins (*Ctsb, Ctsd, Ctss, Ctsz*), glycosidases (*Gusb, Gba, Hexa, Naglu, Man2b1*) and MHCIb genes (*H2-d1, H2-m3, H2-t23, H2-q7*), all upregulated in the Arctic/C5aR1KO (Fig. 6b). *Cd63, Cd68, Ctsl,* and *Gaa* were increased at 2 mo in the Arctic/C5aR1KO relative to the Arctic levels. As with the Cluster 4 genes, representative genes were selected to validate the RNA-seq differential expression by RT-PCR. Genes expression of Cathepsin D and Mannosidase 2b1 were strikingly decreased in the Arctic at 5 months relative to WT (21% and 28% of WT respectively), something not seen in our RNA-seq data, however, both were increased in the Arctic/C5aR1KO relative to the Arctic (6.4× and 4.4× respectively) as seen in our RNA-seq analysis. NPC intracellular cholesterol transporter 2 (Npc2) expression was also greater in the Arctic/C5aR1KO relative to Arctic (1.4×) although no significant difference was seen between the WT and Arctic at 5 months (Fig. 6c, left). At 10 months of age, both Ctsd and Npc2 were upregulated in the Arctic relative to WT, however, only Npc2 was further increased in the Arctic/C5aR1KO (Fig. 6c, right).

### Loss of neuronal complexity in the Arctic model is rescued in the absence of C5aR1

**Fig. 7:**
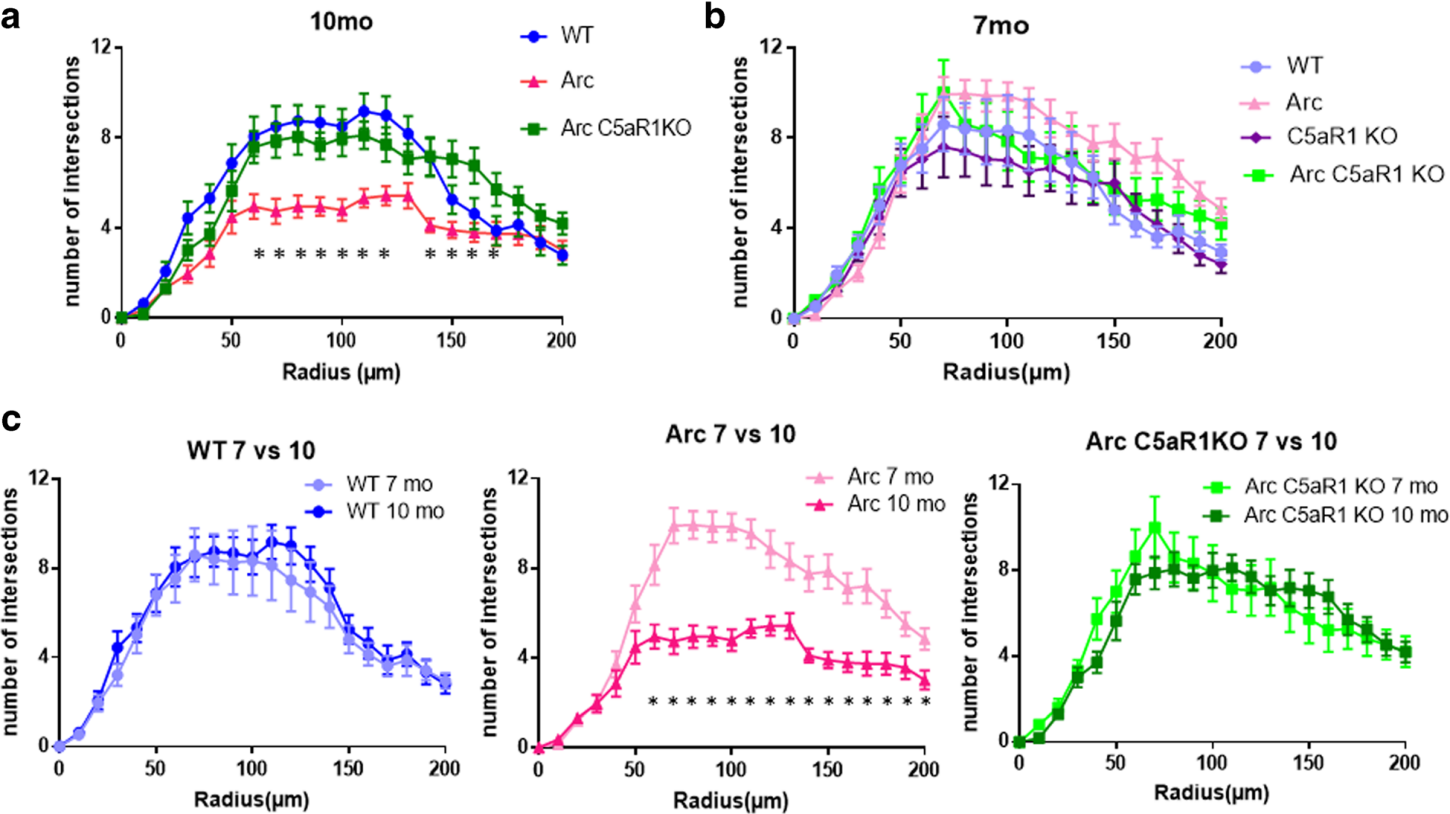
Loss of neuronal complexity in the Arctic model is rescued in the absence of C5aR1. The average number of branchings for a given distance from the soma (um) of each neuron by Sholl analysis using the NeuroLucida software within the CA1 area of 10 mo (**a**) and 7 mo (**b**) mice (5 neurons/ animal, 3 mice per genotype) +/− SEM. * *p* < .05 for Arctic vs Arctic/C5aR1KO by 2 way ANOVA followed by Tukey’s multiple comparison test. (**c**) The same data are plotted to compare the 7 and 10 mo branching in wild type (left), Arctic (middle) and Arctic/C5aR1KO (right) mice, *p < .02 by one way ANOVA

To assess the effect of the C5aR1-deficient environment on neurons, Golgi staining was performed and individual neurons in the CA1 region of the hippocampus were traced and analyzed for the number of branches at 10 μm distances from the cell body. At 10 months of age the number of neuronal branches was significantly reduced in the Arctic mouse relative to the wild type, while the absence of C5aR1 in the Arctic model prevented the decrease, with Arctic/C5aR1KO similar to the wild type in this measure of neuronal complexity (Fig. 7a). This deficit correlated with loss of cognitive performance at 10 months of age (Fig. 1e) as at 7 month of age when no behavior differences are detected (Fig. 1d), neuronal branching in all animals were similar to each other (Fig. 7b). Only the Arctic mouse showed a decline in neurite branching between 7 month and 10 month of age (Fig. 7c). The rescue of the neuronal complexity in the CA1 area of the hippocampus in the Arctic/C5aR1KO aligns with the improvement in the OLM behavior test (hippocampal dependent) in this genotype compared to the Arctic.

## Discussion

Genetic deletion of C5aR1 in the Arctic Alzheimer’s disease mouse model resulted in protection from loss of neuronal complexity in CA1 neurons and prevented deficits in a hippocampal dependent spatial memory task relative to the C5aR1 sufficient Arctic animals. Significant and substantial loss of neuronal complexity and behavior deficits were pronounced at 10 months of age in the Arctic mouse. However, the gene expression profile of the microglia from Arctic/C5aR1KO brain showed a decrease in inflammatory pathways and greater induction of clearance and degradation pathways relative to the Arctic as early as 2–5 months of age. This gene expression profile indicates a polarization state of the microglia that is less inflammatory and more phagocytic that may prevent or delay behavior deficits. Such a timeline of events is consistent with current thought that the disease process starts considerably prior to clinical signs of the disorder in humans. Consequently, treatment to suppress those detrimental processes may best be initiated substantially prior to clinical presentation.

Our previously published studies using a pharmacological approach to block function of C5aR1 in the Tg2576 and 3xTg AD mouse models [7] resulted in reduced fibrillar plaques and gliosis surrounding the plaques. In contrast, the genetic approach to eliminate C5aR1 function used in this Arctic model of AD did not result in reduced fibrillar plaque or microglial accumulation. However, this may due to the “Arctic” mutation in the Aß peptide that leads to rapid fibril formation upon generation of Aß [53]. These fibrils are highly resistant to degradation/clearance. Importantly, the prevention of cognitive loss is seen even in the presence of massive plaque accumulation, supporting the idea that neither the fibrillar plaque itself, nor global microglial markers per se are the major contributor to cognitive loss. Rather it is the specific inflammatory response to the plaque that leads to neurotoxicity [54], perhaps through activation of astrocytes as recently reported [55]. This preservation of cognitive performance in the presence of plaques was recently reported by Lemere and colleagues in another AD model in which C3 had been genetically ablated [16].

TLRs, other innate immune system sensors of danger/damage, when triggered have been found to synergize with C5a-C5aR1 signaling leading to greater proinflammatory cytokine release in models of inflammatory disease in the periphery (reviewed in [56]). Fibrillar Aß is a ligand for TLR2 and TLR4 [57–59] and these receptors have been shown to contribute to the detrimental effects of Aß. C5a may synergistically enhance microglial polarization to a more inflammatory state in response to Aß interactions (or other danger-associated signals) with such receptors on plaque associated glia cells [60]. The increase in NFκB subunits may contribute to increased NF-κB signaling (Fig. 5) and greater expression of inflammatory cytokines and chemokines, all of which were seen in the Arctic mice, but not so elevated in the Arctic/C5aR1KO. In the literature, C5aR1 signaling is most often shown to involve activation of PI-3 K/AKT, PKC, phospholipase D, and MAP kinase (reviewed in [61]). However, instances of NF-κB activation by C5a have also been demonstrated in both human monocytes [62] and macrophages [63]. IL-1, Vegfa, Ccl3, Ccl4, Cxcl2 and Vcam1 are all regulated by the canonical NF-κB (p50/p65) signaling pathway, which can be activated via TLR4 [64]. It is likely that the increase in inflammatory components via NF-κB that we observed is due to a synergy between TLRs and C5aR1, since in the absence of C5aR1 the expression of several inflammatory genes is limited, even though fibrillar amyloid plaque, a TLR ligand, is unchanged.

Also consistent with differential polarization of the microglia in Arctic vs Arctic/C5aR1KO is the increase in phagosomal and lysosomal genes (Fig. 6). Phagocytosis-promoting receptors on microglia such as TREM2, FcγR’s, TLR’s and scavenger receptors have been associated with the clearance of amyloid beta in AD (reviewed in [65]). These genes showed higher expression in the Arctic/C5aR1KO either early in the disease (TREM2, TYROBP, Fcgr4) or later in the disease (TLR2, Colec12). Recent data suggest TREM2 is important in microgliosis and containment of Aß plaques over the progression of the disease [66, 67]. Gene network analysis by Zhang and colleagues identified a microglia specific module that includes TYROBP, the signaling adaptor protein of TREM2, as a key node of networks in late onset Alzheimer’s disease [10].

Cathepsins, proteases that are involved in the endosomal-lysosomal pathway, were substantially and significantly increased in Arctic/C5aR1KO microglia relative to Arctic. Microglial cathepsins, particularly cysteine and aspartyl proteases, have been found to be upregulated with aging in rats (reviewed in [68]), though we did not see an increase in expression in our wild type mice from 2 to 10 months of age. Cathepsin B and D are Aß-degrading enzymes that have been implicated in clearance of amyloid beta in vivo (reviewed in ([69]). Early “activation” of microglia cells has been found to be beneficial in AD models, however, as Aß plaques form, the phagocytosis and clearance of Aß is reduced [70]. Hickman and colleagues found that gene expression of scavenger receptors and degradation enzymes decreased with age in AD mice [71]. C5aR1 deficiency prevents this decrease in phagocytosis and degradation enzymes seen relative to the aging AD mouse models. As mentioned above, fibrillar plaque load in this model is not decreased likely due to the inherent Arctic mutation that accelerates formation of amyloid protofibrils and fibrils, forms of amyloid resistant to ingestion and degradation. However, it is possible that toxic products could still be cleared resulting in the beneficial effects on behavior in the Arctic/C5aR1KO. Cheng and colleagues [31] demonstrated that the Arc48 model used here does show substantial levels of oligomeric (56kD) amyloid and thus it may be the clearance of these forms of Aß and/or neuronal blebs or apoptotic cells are enhanced in the Arctic/C5aR1KO. However, in the more general human AD population that does not have this APP mutation inducing rapid fibrillarization, the functional consequence of this differential gene expression may result in slower plaque accumulation as seen in the 3xTg and Tg2576 AD mouse models when treated with the C5aR1 antagonist, PMX205 [7]. Further investigation will be needed to determine whether or not the differential gene expression in inflammatory components or in clearance capacity or both contribute to the rescue of cognition.

It has been reported that CX3CR1 and CCR2 play a role in the pathogenesis of AD in some mouse models of the disease, either by affecting microglia or by limiting the infiltration of monocytes [49–51]. In the Arctic model we found no difference in amyloid plaque load in mice that possessed a single CX3CR1 or CCR2 allele compared to mice that had both copies of CX3CR1 or CCR2. In addition, no RFP+ myeloid cells were found in the parenchyma, though they were seen around the meninges and were observed (2–6% of FACS-sorted CX3CR1+ cells) by FACS analysis of whole brain suspensions. Given that RFP is expressed in the cytosol when the CCR2 promoter is activated (as in peripheral myeloid cells), and the estimated half-life of RFP in vivo is ~4.6 days [72], we would expect to visualize at least a fraction of RFP+ cells by immunohistochemistry at one of the ages analyzed if indeed CCR2+ monocytes were infiltrating the CNS. The difference between reported observations may be attributed to the different animal models used, which includes different kinetics of accumulation of various species of Aß among other differences.

Inflammation, as mentioned above, is recognized as having a complex role in the pathogenesis of AD (reviewed in [54]). The complement cascade is one aspect of the inflammatory response in AD, which results in both beneficial and detrimental outcomes and synergizes with other innate pathways (reviewed in [73]). Furthermore, epidemiologic and genetic evidence supports a role for complement in the etiology of late onset AD [74], although the GWAS CR1 protein likely has a primary role outside of the CNS on clearance of Aß [75, 76]. The consequences of genetic deletion of C5aR1 reported here support the contention that the mechanism by which the previously used small molecular weight cyclic hexapeptide C5aR1 antagonist, PMX205, was mediating its beneficial effects in AD mouse models [7] was via the inhibition of C5aR1 cell signaling, and support the continued assessment of C5aR1 antagonists for AD therapy. Such specific antagonists have been successfully utilized in acute and chronic inflammatory disease animal models such as sepsis, asthma, ischemia/reperfusion injury, and neurodegenerative diseases [77–80] and as reviewed in [81, 82]. In addition, blocking C5a in rodent CNS disease models has been shown to be beneficial, including in Huntington’s disease [83, 84], traumatic brain injury [85], and ALS [86].

It is critical to note that several studies have demonstrated beneficial effects of complement in the brain (reviewed in [87]). In vitro, C1q has been shown to induce gene expression critical for neuronal survival and protection against oligomer and fibrillar Aß-induced neuronal death [88, 89]. Selective modulation of complement activation products or their receptors may therefore be a more effective strategy than global C1q or complement inhibition for retaining the neuroprotective, anti-inflammatory and phagocytic functions of complement, while dampening damaging excessive inflammation.

**Fig. 8:**
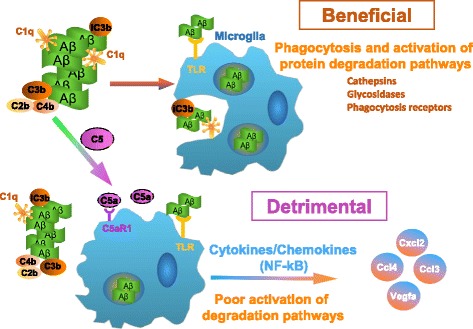
Proposed mechanism by which C5aR1 stimulation contributes to AD progression. Aβ fibrils activate complement resulting in cleavage of C3 and C5. Amyloid deposited C3b/iC3b should lead to more efficient phagocytosis by microglia/macrophages, while generated C5a recruits and alters the function of microglia. C5a induces an inflammatory polarization of the microglia, potentially synergizing with TLRs, which results in production of neurotoxic inflammatory products while suppressing beneficial phagocytosis and degradation pathways thereby contributing to neuronal damage and cognitive deficits. Targeting C5a and its receptor C5aR1 will not interfere with the beneficial effects of upstream complement components

Given the inflammatory profile seen in microglia from Arctic mice with aging and the decrease in expression of the same genes in the Arctic/C5aR1KO, along with the activation of phagocytosis and protein degradation in the Arctic/C5aR1KO, we propose a model by which C5aR1 activation contributes to AD progression (Fig. 8). Complement is activated by fibrillar amyloid beta via the classical (or alternative) pathway and can have beneficial and detrimental effects. Phagocytosis-promoting components such as C1q, C4b, C3b, and iC3b (the latter 3 covalently bound via a thioester bond to plaques) could enhance clearance of Aß fibrils or protofibrils via microglial phagocytosis and/or in the periphery via the potential immune adherence mechanism [76, 90]. Continued downstream activation of the complement cascade leads to the generation of C5a which in the CNS can bind to microglial C5aR1 and synergize with TLR’s when the microglia engage a plaque, polarizing the microglia to a more inflammatory state and suppressing upregulation of phagosomal and degradation pathways. The resulting inflammatory environment further damages neurons, perhaps via astrocyte activation [55], thereby contributing to cognitive deficits. If this model is correct, targeting C5aR1 will not disrupt the beneficial effects of the upstream complement activation products, but rather, it will suppress polarization of the microglia to an inflammatory state, allowing the phagocytosis and degradation promoting gene expression program of the cells to continue. Further in depth analysis is needed to test this proposed model.

Recent elegant studies have shown that excessive complement-mediated synapse pruning occurs in aging, AD, and other disorders leading to cognitive or behavior changes [17, 18, 91, 92]. Since the activation of complement in these scenarios could also result in the generation of C5a, it remains to be seen if it is the insult of C5a-mediated effects that directly results in the behavioral/cognitive dysfunction or if C5aR1 signaling influences synaptic pruning at the microglial or neuronal level, or if both mechanisms contribute independently to deficits. While additional studies in multiple animal models can be performed to discern this, what the balance is in human systems will require clinical trials. The lack of detrimental toxicity of the C5aR1 antagonist PMX53 and CCX168 in human clinical trials of refractory rheumatoid arthritis [93] and ANCA-associated vasculitis [94], and the clinical experience with FDA approved Eculizumab, an anti C5 monoclonal antibody that prevents the cleavage of C5 and thus the generation of C5a [95], suggests that suppression of C5a/C5aR1 signaling may not be harmful for extended clinical use in adults. In addition to the potential for greater access to the brain, an advantage of a selective C5aR1 antagonist vs Eculizumab for long term treatment of diseases such as AD is that, while it blocks effects of C5a, the generation of C5b by the uninhibited cleavage of C5 is still able to initiate the assembly of the bacteriolytic C5b-9 complex upon complement activation by pathogens. Thus, this protective bacteriolytic function of complement would not be systemically compromised by the antagonist as it is by Eculizumab. The preservation of CA1 neuronal complexity seen here provides proof of concept that blocking C5a-C5aR1 could prevent detrimental downstream effects of the complement cascade activation and thereby suppress damage to neurons, while preserving systemic protection from infection, which could be compromised by targeting other components in the complement cascade as treatment for this disease.

## Conclusion

Genetic ablation of C5aR1 in a mouse model of AD resulted in microglial gene expression profile indicative of less inflammation and greater induction of clearance pathways, preserved neuronal complexity in the hippocampus and prevented deficits in a hippocampal dependent spatial memory task. These data, consistent with the increasingly proposed role of inflammation in neurodegenerative disorders, provide additional compelling rationale to pursue inhibition of C5aR1 as a strategic targeted therapy to slow the progression of AD in humans.

## Additional files

Additional file 1: Representative habituation performance during testing and performance during training. Representative analysis of (A) Speed (B) distance traveled (C) percent of total time spent in an inner zone and (D) percent of total time spent in an outer zone quantified using the ANY-maze software. Data are from 10 months animals: *n* = 19 (WT), 16 (C5aR1KO), 7 (Arctic) and 8 (Arctic/C5aR1KO), representative of at least 2 experiments and similar assessments in 7 months animals. (E) During training, all genotypes were assessed for time spent with each object (left and right of the context). Bars show average percent time +/− SEM per object (left and right) for each genotype at 7 months. *n* = 10, 9, 9, and 10 for WT, C5aR1KO, Arctic, and Arctic/C5aR1KO respectively. No preference for left or right is detected by one- way AVOVA. (DOCX 241 kb)

Additional file 2: Comparison of IBA1 microglia and Thioflavine S plaque size between Arctic and Arctic/C5aR1KO. (A-D) Representative images of the hippocampus stained for IBA1 and ThioS. (E) Quantification of the number of IBA1+ cells/field of view (FOV) using the spots modules in Bit-plane Imaris 7.5. (F) Quantification of the size of ThioS+ plaques/FOV using the surfaces module in Bit-plane Imaris. Error bars represent SEM, (*n* = 3–4/group). *p < 0.05. Scale bar in D = 100 μm. (DOCX 113 kb)

Additional file 3: Aβ plaque load and CD45 expression in Arctic heterozygous for CX3CR1 and CCR2. Brain sections from Arctic, Arctic-CX3CR1^+/GFP^ or Arctic-CCR2^+/RFP^ reporter mice at 6 or 7 months were stained with either thioflavine (A), or an anti-Aß antibody (1536) (C) to assess the plaque load (A, C). CD45 reactivity was probed to investigate microglial activation (B,D). Arctic mice were compared to Arctic mice heterozygous for CCR2-RFP (A, B) or heterozygous for Cx3CR1-GFP (C,D). Scale bar is 100 μm. (E). Bars represent the average Field Area % of 3–4 animals per genotype (2 sections per animal). No statistically significant difference was observed in Arctic compared to Arctic-CCR2^+/RFP^ in thioflavine (*p* < 0.75) or CD45 (*p* < 0.09) or between Arctic and Arctic-CX3CR1^+/GFP^ in Aß (*p* < 0.67) or CD45 (*p* < 0.71) by one-way ANOVA statistical analysis. (DOCX 1787 kb)

Additional File 4: RNA quality control. Amount of RNA extracted from FACS-sorted microglia isolated from adult mice was quantified by NanoDrop and the quality assessed by Agilent Bioanalyzer. All samples had greater than 1 ng/μl of RNA and RINs greater than 4, sufficient for the SMARTer Stranded Total RNA-Seq Kit - Pico Input Mammalian by Clontech. (DOCX 11 kb)

Additional file 5: Dynamic gene expression profiles of 9 clusters. Gene expression clusters were derived from maSigPro. Median profile are plotted across all ages for all the genes in the corresponding cluster. WT (blue), C5aR1KO (red), Arctic (green) and Arctic/C5aR1KO (orange). (DOCX 265 kb)

Additional file 6: Gene ontology enrichment analysis. Clusters were analyzed with Metascape (http://metascape.org) to identify the GO terms (biological processes) enriched in each cluster. The GO terms, their descriptions, logP, log(q-value), InTerm_InList (Genes in cluster/All genes from genome in GO term), and the gene and gene symbols are listed. (XLSX 243 kb)

Additional file 7: Pathway analysis. Significantly enriched KEGG pathways were identified by PaintOmics 3. The pathway name, number of genes in the pathway, and the *p*-value of the enriched pathway is displayed for each cluster. (XLSX 23 kb)

Additional file 8: Genes found in each maSigPro cluster. Genes present in each cluster are listed with Ensembl ID and gene symbol noted. (XLSX 100 kb)

## Abbreviations

AD: Alzheimer’s disease
Aß: Amyloid beta peptide
C5aR1: C5a receptor 1, CD88
FACS: Fluorescence-activated cell sorting
fAß: Fibrillar amyloid beta peptide
GFP: Green fluorescent protein
GO: Gene ontology
HBSS: Hanks’ balanced salt solution
iNOR: Hippocampal independent novel object recognition
KO: Genetic knock out
OLM: Object location memory
PBS: Phosphate buffered saline
PCR: Polymerase chain reaction
RFP: Red fluorescent protein
SEM: Standard error mean
TBS: Tris-buffered saline
TLR: Toll-like receptor
TPM: Transcripts per million
WT: Wild type
