# Supplementary material for: Prevention of C5aR1 signaling delays microglial inflammatory polarization, favors clearance pathways and suppresses cognitive loss
Source: Mol Neurodegener. 2017 Sep 18;12:66. doi: 10.1186/s13024-017-0210-z (PMC5604420; doi:10.1186/s13024-017-0210-z)
Supplement: Supplementary file 1 — Representative habituation performance during testing and performance during training. Representative analysis of (A) Speed (B) distance traveled (C) percent of total time spent in an inner zone and (D) percent of total time spent in an outer zone quantified using the ANY-maze software. Data are from 10 months animals: n = 19 (WT), 16 (C5aR1KO), 7 (Arctic) and 8 (Arctic/C5aR1KO), representative of at least 2 experiments and similar assessments in 7 months animals. (E) During training, all genotypes were assessed for time spent with each object (left and right of the context). Bars show average percent time +/− SEM per object (left and right) for each genotype at 7 months. n = 10, 9, 9, and 10 for WT, C5aR1KO, Arctic, and Arctic/C5aR1KO respectively. No preference for left or right is detected by one- way AVOVA. (DOCX 241 kb) [file 13024_2017_210_MOESM1_ESM.docx]

**Supplemental Materials**


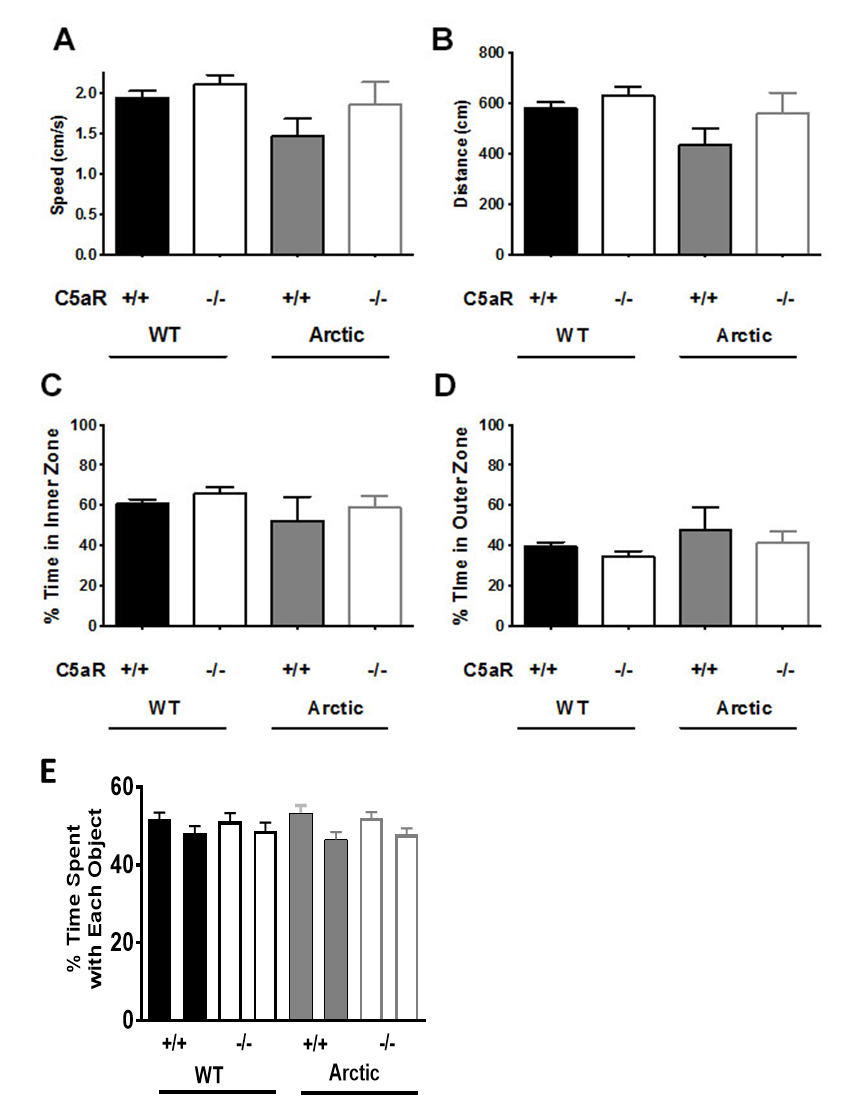


**Additional File 1**

**Representative habituation performance during testing and performance during training.**

Representative analysis of **(A**) Speed (**B)** distance traveled **(C)** percent of total time spent in an inner zone and **(D)** percent of total time spent in an outer zone quantified using the ANY-maze software. Data are from 10 months animals: n = 19 (WT), 16 (C5aR1KO), 7 (Arctic) and 8 (Arctic/C5aR1KO), representative of at least 2 experiments and similar assessments in 7 months animals. **(E)** During training all genotypes were assessed for time spent with each object (left and right of the context). Bars show average percent time +/- SEM per object (left and right) for each genotype at 7 months. n = 10, 9, 9, and 10 for WT, C5aR1KO, Arctic, and Arctic/C5aR1KO respectively. No preference for left or right is detected by 1 way AVOVA.
