# Supplementary material for: Prevention of C5aR1 signaling delays microglial inflammatory polarization, favors clearance pathways and suppresses cognitive loss
Source: Mol Neurodegener. 2017 Sep 18;12:66. doi: 10.1186/s13024-017-0210-z (PMC5604420; doi:10.1186/s13024-017-0210-z)
Supplement: Supplementary file 2 — Comparison of IBA1 microglia and Thioflavine S plaque size between Arctic and Arctic/C5aR1KO. (A-D) Representative images of the hippocampus stained for IBA1 and ThioS. (E) Quantification of the number of IBA1+ cells/field of view (FOV) using the spots modules in Bit-plane Imaris 7.5. (F) Quantification of the size of ThioS+ plaques/FOV using the surfaces module in Bit-plane Imaris. Error bars represent SEM, (n = 3–4/group). *p < 0.05. Scale bar in D = 100 μm. (DOCX 113 kb) [file 13024_2017_210_MOESM2_ESM.docx]

**
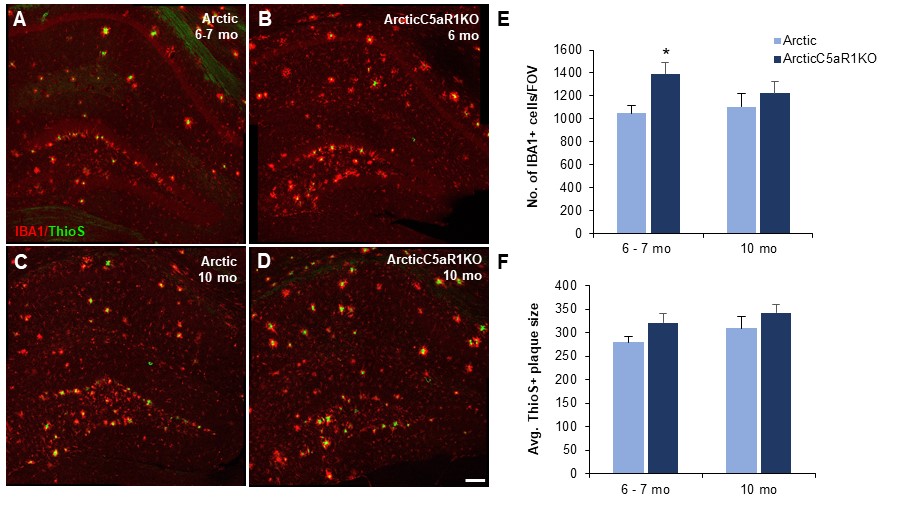
**

**Additional file 2. Comparison of IBA1 microglia and Thioflavine S plaque size between Arctic and Arctic/C5aR1KO.** (A-D) Representative images of the hippocampus stained for IBA1 and ThioS. (E) Quantification of the number of IBA1+ cells/field of view (FOV) using the spots modules in Bit-plane Imaris 7.5. (F) Quantification of the size of ThioS+ plaques/FOV using the surfaces module in Bit-plane Imaris. Error bars represent SEM, (n=3-4/group). *p < 0.05. Scale bar in D = 100 µm.
