# Supplementary material for: Prevention of C5aR1 signaling delays microglial inflammatory polarization, favors clearance pathways and suppresses cognitive loss
Source: Mol Neurodegener. 2017 Sep 18;12:66. doi: 10.1186/s13024-017-0210-z (PMC5604420; doi:10.1186/s13024-017-0210-z)
Supplement: Supplementary file 3 — Aβ plaque load and CD45 expression in Arctic heterozygous for CX3CR1 and CCR2. Brain sections from Arctic, Arctic-CX3CR1+/GFP or Arctic-CCR2+/RFP reporter mice at 6 or 7 months were stained with either thioflavine (A), or an anti-Aß antibody (1536) (C) to assess the plaque load (A, C). CD45 reactivity was probed to investigate microglial activation (B,D). Arctic mice were compared to Arctic mice heterozygous for CCR2-RFP (A, B) or heterozygous for Cx3CR1-GFP (C,D). Scale bar is 100 μm. (E). Bars represent the average Field Area % of 3–4 animals per genotype (2 sections per animal). No statistically significant difference was observed in Arctic compared to Arctic-CCR2+/RFP in thioflavine (p < 0.75) or CD45 (p < 0.09) or between Arctic and Arctic-CX3CR1+/GFP in Aß (p < 0.67) or CD45 (p < 0.71) by one-way ANOVA statistical analysis. (DOCX 1787 kb) [file 13024_2017_210_MOESM3_ESM.docx]

**
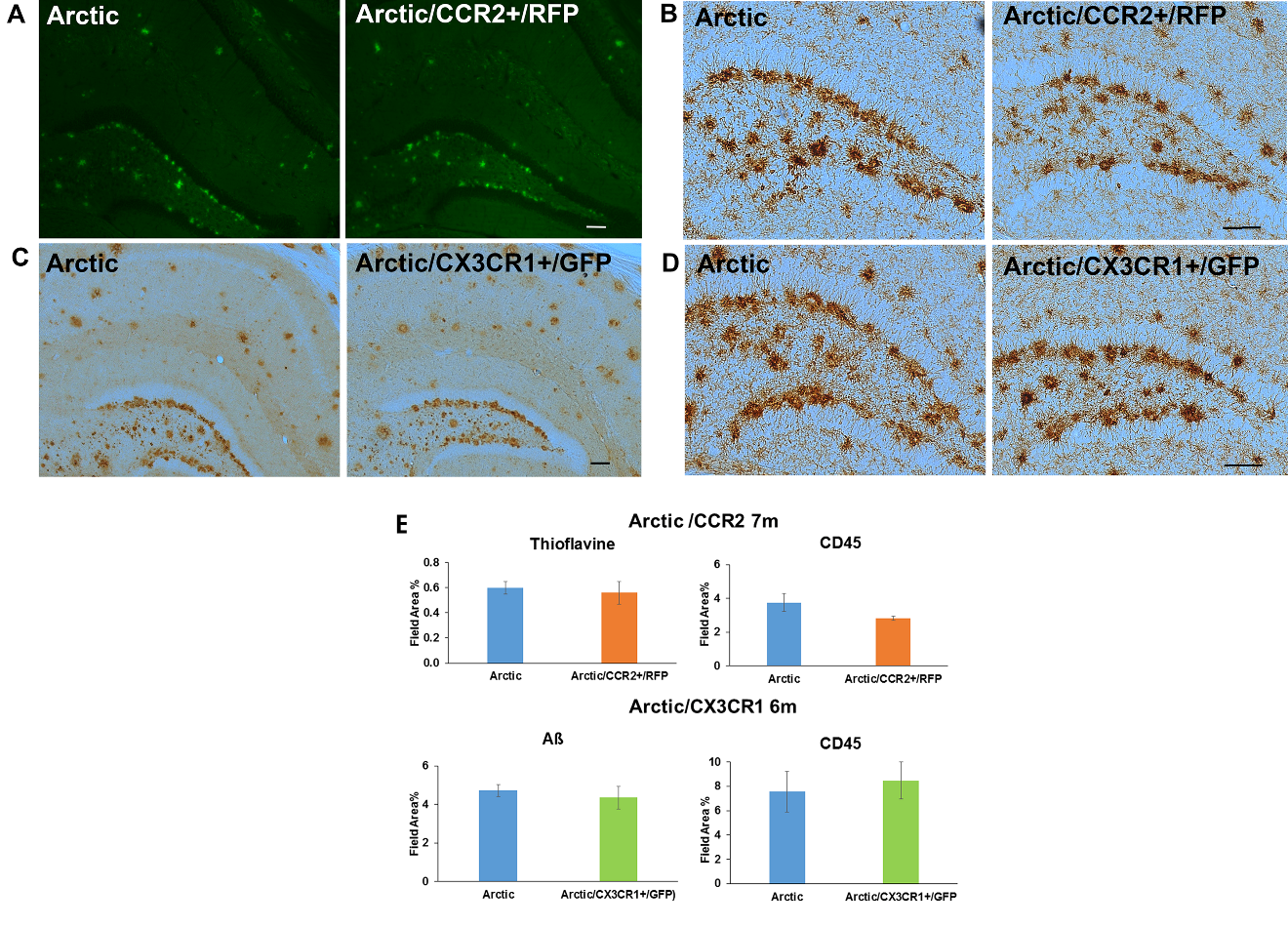
Additional file 3. Aβ plaque load and CD45 expression in Arctic heterozygous for CX3CR1 and CCR2.** Brain sections from Arctic, Arctic-CX3CR1^+/GFP^ or Arctic-CCR2^+/RFP^ reporter mice at 6 or 7 months were stained with either thioflavine (**A**), or an anti-Aß antibody (1536) (**C**) to assess the plaque load (**A, C**). CD45 reactivity was probed to investigate microglial activation (**B,D**). Arctic mice were compared to Arctic mice heterozygous for CCR2-RFP (**A, B**) or heterozygous for Cx3CR1-GFP (**C,D**). Scale bar is 100 µm. (**E**). Bars represent the average Field Area % of 3-4 animals per genotype (2 sections per animal). No statistically significant difference was observed in Arctic compared to Arctic-CCR2^+/RFP^ in thioflavine (p<0.75) or CD45 (p<0.09) or between Arctic and Arctic-CX3CR1^+/GFP^ in Aß (p<0.67) or CD45 (p<0.71) by one-way ANOVA statistical analysis.
