# Supplementary material for: Prevention of C5aR1 signaling delays microglial inflammatory polarization, favors clearance pathways and suppresses cognitive loss
Source: Mol Neurodegener. 2017 Sep 18;12:66. doi: 10.1186/s13024-017-0210-z (PMC5604420; doi:10.1186/s13024-017-0210-z)
Supplement: Supplementary file 4 — RNA quality control. Amount of RNA extracted from FACS-sorted microglia isolated from adult mice was quantified by NanoDrop and the quality assessed by Agilent Bioanalyzer. All samples had greater than 1 ng/μl of RNA and RINs greater than 4, sufficient for the SMARTer Stranded Total RNA-Seq Kit - Pico Input Mammalian by Clontech. (DOCX 11 kb) [file 13024_2017_210_MOESM4_ESM.docx]

**Additional File 4**

| **Genotype** | **Age** | **[RNA] ng/µL** | **RIN** |
| --- | --- | --- | --- |
| WT | 2 | 8 - 10 | 4.8 - 7.3 |
| WT | 5 | 5 - 6 | 6.0 - 7.4 |
| WT | 7 | 6 - 7 | 6.8 - 8.3 |
| WT | 10-11 | 7 - 10 | 7.6 - 8.1 |
| C5aR1KO | 2 | 5 | 7.6 - 7.8 |
| C5aR1KO | 5 | 8 | 7.9 - 8.4 |
| C5aR1KO | 7 | 1 - 2 | 8.5 - 8.8 |
| C5aR1KO | 10-11 | 10 - 12 | 8.5 - 8.9 |
| Arctic | 2 | 9 - 11 | 4.1 - 5.6 |
| Arctic | 5 | 10 - 16 | 6.7 - 7.0 |
| Arctic | 7 | 6 - 7 | 7.7 - 8.1 |
| Arctic | 10-11 | 9 - 10 | 8.2 - 9.3 |
| Arctic/C5aR1KO | 2 | 4 - 5 | 7.1 - 7.8 |
| Arctic/C5aR1KO | 5 | 1 - 4 | 7.7 - 8.0 |
| Arctic/C5aR1KO | 7 | 1 - 2 | 8.8 - 9.2 |
| Arctic/C5aR1KO | 10-11 | 8 - 11 | 9.2 - 9.5 |

**Table S1. RNA QC.** Amount of RNA extracted from FACS-sorted microglia isolated from adult mice was quantified by NanoDrop and the quality assessed by Agilent Bioanalyzer. All samples had greater than 1ng/µl of RNA and RINs greater than 4, sufficient for the SMARTer Stranded Total RNA-Seq Kit - Pico Input Mammalian by Clonetech.
