# Supplementary material for: Prevention of C5aR1 signaling delays microglial inflammatory polarization, favors clearance pathways and suppresses cognitive loss
Source: Mol Neurodegener. 2017 Sep 18;12:66. doi: 10.1186/s13024-017-0210-z (PMC5604420; doi:10.1186/s13024-017-0210-z)
Supplement: Supplementary file 5 — Dynamic gene expression profiles of 9 clusters. Gene expression clusters were derived from maSigPro. Median profile are plotted across all ages for all the genes in the corresponding cluster. WT (blue), C5aR1KO (red), Arctic (green) and Arctic/C5aR1KO (orange). (DOCX 265 kb) [file 13024_2017_210_MOESM5_ESM.docx]

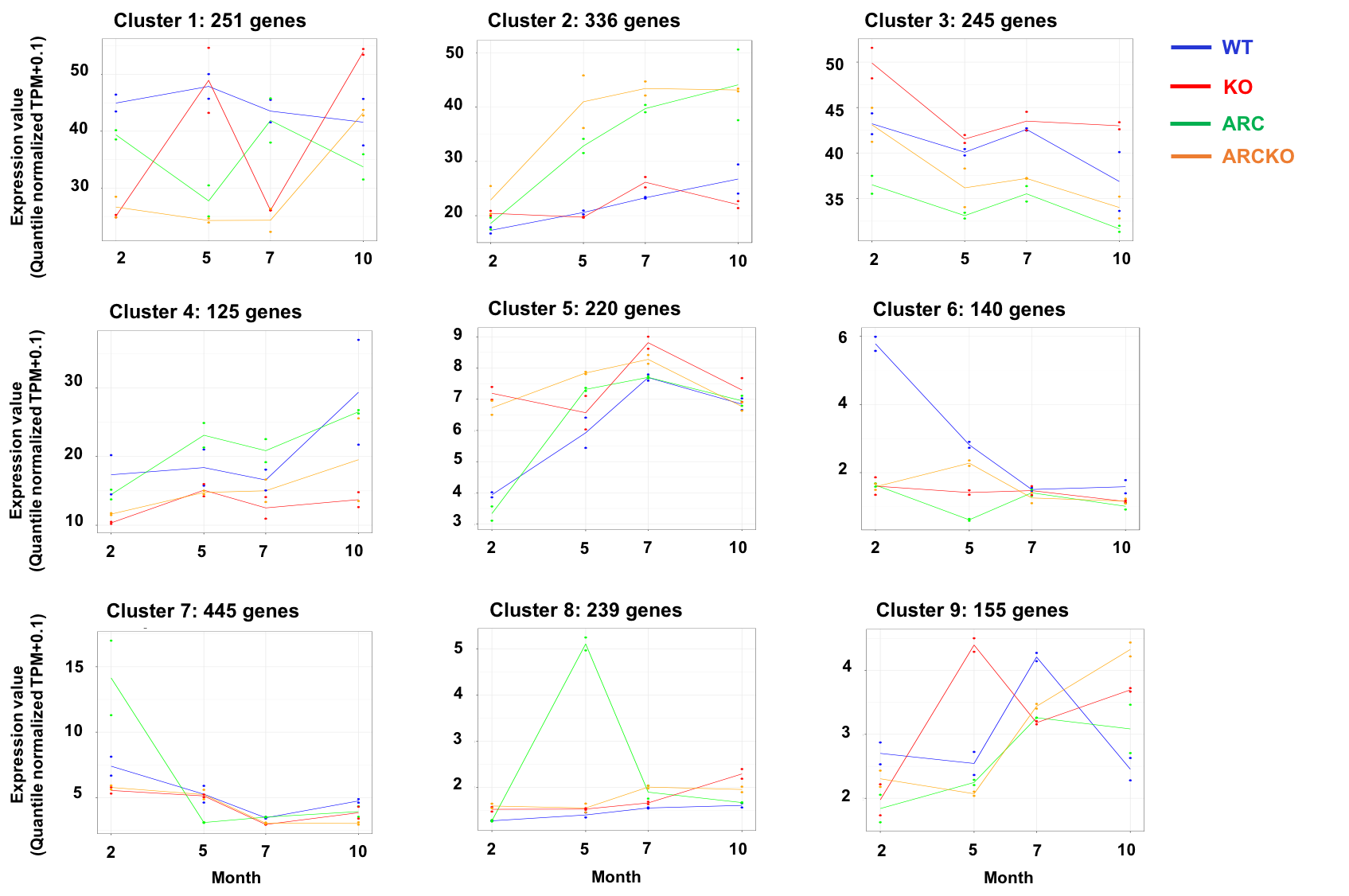


**Additional file 5. Dynamic gene expression profiles of remaining 7 clusters.** Gene expression clusters were derived from maSigPro. Median profile are plotted across all replicates for all the genes in the corresponding cluster. WT (blue), C5aR1KO (red), Arctic (green) and Arctic/C5aR1KO (orange).
